# The Quest for Contrast in Digital Images of Micro/Nano Structured Polymer Blends Before and After CO_2_ Foaming: Impact of Block Copolymer Content and Size Upon Acrylic Cellular Structures

**DOI:** 10.1002/smll.202405730

**Published:** 2024-12-23

**Authors:** Yannick Anguy, Margaux Haurat, Michel Dumon

**Affiliations:** ^1^ UMR CNRS 5295 laboratoire I2M Université de Bordeaux Talence F‐33405 France; ^2^ UMR CNRS 5629, laboratoire LCPO Université de Bordeaux Pessac F‐33600 France

**Keywords:** acrylic polymers and copolymer, contrast and edge enhancement, materials morphological characterization, micron and nano sized foams, one‐step batch scCO_2_ foaming

## Abstract

This work addresses the structural quantification of multiphase materials, here nanostructured polymer solid precursors and their micro/nano sized foamed counterparts. It is based on a strategy of contrast/edge enhancement, locally adaptive to image data in digital images of materials. The method allows to binarize straightforwardly the structures (the phases) in TEM and SEM images after edge identification, edge choice, and image virtual reconstruction. A detailed insight is brought into one‐step batch supercritical CO_2_ foaming of acrylic amorphous PMMA (polymethyl methacrylate) polymers, aided by the nanostructuration of block copolymers (BCP), here MAM (butyl acrylate center block methyl acrylate side blocks). The foaming conditions, i.e., pressure drop rate (PDR) and saturation temperature required for an *actual* one‐step procedure are specified and clarified, whereas previous works, dealing with “one‐step procedures”, are probably incurring in a two‐step procedure. The roles of the BCP content (and size) and saturation temperature are carefully analyzed and further clarified, more comprehensively than in previous literature. Thanks to the analysis of size distributions of foams and foam blend precursors (0.25, 0.5, 10 wt% MAM), bi modality of 10 wt% foams is for example revealed. A discussion of kinetics effects, i.e., evolutions of the effective sample temperature *T_ef_* (*t*), and the effective glass transition temperature *T*_*g*, *ef*_(*t*). provides a new insight of “pseudo” one‐step VS “real” one‐step batch foaming.

## Graphical abstract

Analysis of morphologies via a non linear transient (time‐dependent) diffusion process of an acrylic bimodal FOAM from a nano structured blend MAM10 wt%/PMMA90 wt%.

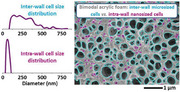

## 1 Introduction

Over the last decade, micro‐/nano sized polymer foams, in particular those produced by means of a physical blowing agent, have become popular. Indeed, polymeric foams with reduced porosity dimensions can offer an advantageous combination of improved properties, e.g., a very low thermal conductivity and/or reinforced mechanical properties in terms of damping versus rigidity and also toughness^[^ ^1^, ^2^, ^3^, ^4^ ^]^ Yet, producing *nanosized* foams using a *physical blowing agent* is still a challenge, which requires fine‐tuning of the foaming conditions for each combination of foaming agent – material – process. References^[^ ^5^, ^6^, ^7^, ^8^ ^]^ summarize the main challenges and strategies for nano – micro foaming with a pionneering work using fluorinated block copolymers cast films.^[^ ^6^ ^]^ The mastering and understanding of the foaming mechanism (/and process) VS material structure is the key for controlling the final porous morphologies and properties. The morphologies (before and after foaming), at micro and nano levels, should therefore be well characterized and analyzed all along the process. A proper analysis of the morphologies [even more difficult in “all organic” multiphase materials, as polymer blends or dispersions] requires a suitable contrasting method, imaging in proper conditions (temperature, pressure, electron flow) and, finally, establishing robust methods with refined image treatments.

Anguy et al.^[^ ^9^ ^]^ thoroughly discussed a method in the analysis of images and morphologies to identify and segment the right interfaces and interphases for a sound analysis of the structuration of organic multiphase materials at the micro and nano scales. The result is an *image virtual reconstruction* where objects’ boundaries are adaptably and selectively enhanced (contrasted) for a simple subsequent segmentation of phases.

This methodology is here originally applied to acrylic polymers in two different states: solid state polymer blends (acting as foam precursors, with isolated nanodomains), then the resulting scCO_2_ (supercritical CO_2_) blown foams, with either nano or micro cells. Note that the definition of “nano” and “micro” in the field of polymer foams is intended as follows: “nano cellular” is used for voids (cells, pores) below 300 nm, “micro” for voids between 1000 nm to 100 microns, “macro” for voids bigger than ∼100 microns, “ultramicro cellular” between 0.3 and 1 micron (note that this definition differs from the inorganic field's definition (IUPAC definition).

Carbon dioxide (CO_2_) is here used as a physical blowing agent representing a green alternative to chemical foaming agents, e.g., azodicarbonamide (ADA), which use is being restricted by incoming laws. This non‐toxic and low‐cost foaming agent enters the supercritical state (scCO_2_) in soft conditions (with a critical point at *P_c_* = 7.38 MPa and *T_c_* = 31 °C), in which it combines advantageously liquid and gaseous properties, i.e., a good solubility and a good diffusivity in polymers,^[^ ^10^ ^]^ for an optimized saturation.

High CO_2_‐philicities of acrylics make acrylic polymers good candidates for scCO_2_ foaming.^[^ ^11^ ^]^ Acrylic polymer blends are chosen, for the solid precursors, and composed of a homopolymer matrix, i.e., PMMA, poly (methyl methacrylate) and a dispersed triblock copolymer (BCP), named MAM, poly (methyl methacrylate‐co‐butylacrylate‐co‐methyl methacrylate), 90/10, 99.5/0.5 and 99.75/0.25 PMMA/MAM weight ratios^[^ ^9^, ^12^ ^]^ Due to chemical similarity and covalent bonded blocks, MAM separates only at the nanoscale as micellar‐like objects, or nanostructures, dispersed in the PMMA matrix. Given the higher CO_2_‐philicity of the soft poly (butyl acrylate) (PBA) center block, the nanostructures are deemed to act as CO_2_ enhanced local reservoirs compared to PMMA. They act as efficient nucleants improving PMMA foaming in terms of cell size reduction and homogeneity, e.g., Dumon et al.^[^ ^13^ ^]^

 sc CO_2_ foaming processes include extrusion^[^ ^14^, ^15^ ^]^ injection^[^ ^16^, ^17^ ^]^ and batch foaming^[^ ^7^, ^13^, ^18^ ^]^ Batch foaming, or autoclave foaming, either one‐step or two‐step, is often preferred over the other processes for producing foams with small porosity dimensions.^[^ ^18^ ^]^ The reason for this is that, unlike other processes, the batch scCO_2_ saturation conditions are independently and easily controlled. In this process, after scCO_2_ saturation, phase separation between the polymeric material and the gas is induced by a pressure quench. The resulting thermodynamic instability initiates foaming and the cell nucleation, growth and coalescence steps follow. All three steps may or not partially overlap over time, depending on temperature and pressure evolutions, and kinetics of vitrification.

The structure of the foam depends on the material formulation, e.g., the block copolymer (BCP) content and size and on the foaming parameters. The latter include the saturation temperature *T_sat_* and pressure *P_sat_* and the pressure drop rate (PDR) to trigger foaming. So the final porous structure is influenced by the interplay and competition of the kinetics of several of the intervening variables, i.e., the respective evolution with time *t* of the material effective temperature *T_ef_* (*t*), the effective glass transition temperature *T*_*g*, *ef*_(*t*) of the plasticized system (polymer + CO_2_) and the instantaneous PDR, $\frac{dP(t)}{dt}$, which generally differs from the imposed average PDR, $\frac{\Delta P}{\Delta t}=\frac{P_{sat}-P_{ambient}}{\Delta t}$ (where Δ*t* is the time to return to ambient pressure upon pressure release).

Assessing the impact of the above factors and their respective kinetics is facilitated when **(1)** the BCP nanostructures size distribution of the unfoamed material and **(2)** the cell size distributions of the foam are available (the latter is always influenced by the former).

Within the practical scope of designing functional foams, the structural characteristics derived from representative size distributions such as the average cell size or the cell number density are of great help to correlate the micro‐/nanoporous structure to the physical properties of the foam.^[^ ^19^, ^20^, ^21^, ^22^, ^23^ ^]^

The automatic calculation of the size distribution of an object set (nanostructures or porous cells) first requires to accurately segment, or binarize, the objects sampled in a gray level digital image. This is often tedious and time consuming, since the appropriate image treatment is generally image‐dependent. In this work, the automatic segmentation step is greatly facilitated by preparatorily enhancing the contrast of the object set, sampled in a (series of) electronic micrograph(s). The approach is based on a transient diffusion process whereby the local gray level intensity is allowed to spread across the image pixels. This diffusion process is directional and locally adaptive to image data; the reader may refer to ref. [9]

In this context, the main goal of this work is to address the impact of the saturation temperature *T_sat_* and the BCP content and size upon the final foam structure through a gas foaming batch process. This involves taking advantage of availability of representative statistical size distributions, which accurately quantify the morphology of the foaming process input and output. We successfully provide a new insight on long‐overlooked works using the gas dissolution approach to produce nanosized polymeric foams, in an *actual* one‐step procedure. It is argued that previous attempts using high PDR and/or low saturation temperatures, and presented as batch one‐step gas foaming, were most likely experiencing a two‐step procedure for kinetic reasons.

## 2 Experimental Section

### 2.1 Materials

Pellets of neat poly (methyl methacrylate) (PMMA) commercialized as V825T 101 Clear grade was initially supplied by Arkema Company (Lacq, France) and is now commercialized by Trinseo. This PMMA shows a melt flow index (MFI) of 2.8 g/10 min (measured at 230 °C and 3.8 kg), a density of 1.19 ± 0.01 g cm^−3^ and a glass transition temperature of 115 °C measured by DSC. Its number average molar mass is *M_n_* =  43 kg mol^−1^ and weight average molar mass is *M_w_* =  82 kg mol^−1^.

Pellets of neat MAM (poly (methyl methacrylate)‐co‐poly (butyl acrylate)‐co‐poly (methyl methacrylate)) triblock copolymers are also supplied by Arkema. This copolymer of commercial named Nanostrength M53 has a 54 wt% content of the soft block, poly (butyl acrylate) (PBA). It shows a MFI of 0.208 ± 0.003 g/10 min measured at 160 °C and 10 kg^[^ ^12^ ^]^ and a glass transition temperature of − 40 °C measured by DSC. The molecular weight is *M_n_* =  82 kg mol^−1^ and *M_w_* =  128 kg mol^−1^.

### 2.2 Solid Blends Production

PMMA/MAM blends were compounded using a co‐rotative twin‐screw extruder provided by Labtech, with *L*/*D* ratio of 40 and a screw diameter of 26 mm. Before compounding, neat PMMA and MAM pellets were dried at 80 °C for 4 h in an oven. Then, the blends were extruded at a screw speed of 300 rpm with a temperature profile from 250 °C at the extruder inlet to 230 °C in the die. Last, the blends were pelletized using a continuous cutting machine operating at the end of the line. Blends with three different MAM contents, i.e., 0.25%, 0.5%, and 10 wt% were produced.

PMMA/MAM pellets were dried again at 80 °C for 4 h. Transparent tensile test bars (ISO 180/U 80 × 10 × 4 mm) of the three blends were injected with a classical injection‐molding device (ENGEL ES 200–45 HL‐V). The acrylic blends were injected at 230 °C at a screw speed of 300 rpm in a mold heated at 90 °C. All bulk bars were perfectly transparent.

### 2.3 Gas Dissolution Foaming

Foaming experiments were performed at the LCPO laboratory (Bordeaux, France) in a high‐pressure vessel (provided by TOP Industrie, Vaux‐le‐Pénil, France) with a capacity of 0.3 L, capable of operating at a maximum temperature of 250 °C and a maximum pressure of 40 MPa. Pressure was set to the desired value with a fluid pump (model Teledyne ISCO 260 provided by Teledyne ISCO, Lincoln, USA). The temperature was imposed using a clamp heater connected to a controller. Foaming was performed using a one‐step discontinuous process.^[^ ^22^, ^24^, ^25^ ^]^ Solid samples (80 × 10 × 4 mm) were introduced (and hung) in the vessel under 30 MPa of CO_2_ pressure for the saturation stage. Two different saturation temperatures were used, 40 or 60 °C. Under such operating conditions, the CO_2_ is supercritical. Saturation time was 24 h for the maximum CO_2_ uptake. After saturation, pressure was released at a moderate average PDR of 0.5 MPa s^−1^.

Recall that CO_2_ sorption depends on pressure and temperature. At 30 MPa and 40 °C, the solubility of CO_2_ in PMMA is ≈22 wt%. This value was obtained using a Fourier transform infrared microscope coupled to a high‐pressure cell.^[^ ^26^, ^27^, ^28^ ^]^ Using this method, it was measured that MAM presents a much higher CO_2_‐affinity than PMMA^[^ ^29^, ^30^, ^31^ ^]^ and can absorb ≈45 wt% at 30 MPa and 40 °C.

### 2.4 Characterization Methods

#### 2.4.1 Density

The density of the solid blends (ρ_*s*_) and the foamed materials (ρ_*f*_) was determined by water displacement, based on Archimedes’ principle. For each sample, three measurements were made. The solid skin of the foams was very thin and insignificant for the density measurement. This has been verified for early produced foams for which density values measured from polished and raw samples were fully comparable. In view of this, the solid skin was not removed in all subsequent density measurements.

#### 2.4.2 Nanostructuration of the Solid Blends

The structure of the solid PMMA/MAM blend precursors was analyzed by transmission electron microscopy (TEM). Each material was first cut into thin slices with a thickness of ≈80 nm using a Leica EM UC7‐FC7 ultra microtome. Due to the soft behavior of the acrylic blend, samples were cut in cryogenic conditions at − 75 °C. Slices were next collected and laid down onto 200 mesh copper TEM grids.

Prior to TEM observation, the thin cuts were stained during 10 min at room temperature by an aqueous solution of 2 wt% of phosphotungstic acid (PTA) and 2 wt% of benzyl alcohol in order to increase contrast between the PMMA matrix and the dispersed MAM nanostructures. As reported in the literature, PTA preferentially colors at room temperature the CO_2_‐philic poly (butyl acrylate) (PBA) soft block of MAM (in dark gray in TEM images) over the PMMA more rigid blocks (in light gray in TEM images)^[^ ^32^, ^33^, ^34^, ^35^ ^]^ Benzyl alcohol acts as a dyeing assistant and further helps coloration of PBA by PTA.

**Figure 1:**
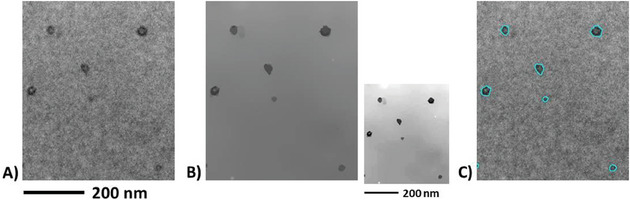
A) TEM micrograph (local area; magnification: 120 kX) illustrating the nanostructuration (darker spots) of a PMMA/MAM blend (0.25 wt% MAM) after staining by PTA. B) Piecewise constant image representing a simplified image at a lower resolution with sharper boundaries after running over Figure 1A a nonlinear diffusive filter.^[^ ^9^ ^]^ In order to better appraise the beneficial action of the nonlinear filter, the small inset shows Figure 1B after post processing by standard histogram stretching. C) Segmented counterpart of Figure 1B by simple thresholding of the gray level histogram. The level set corresponding to the threshold value is superimposed in cyan upon the original image (Figure 1A).

TEM images were collected at the Bordeaux Imaging Center (Bordeaux France) with a Hitachi H7650 electron microscope equipped with a camera SC1000 ORIUS 11Mpx (GATAN). The acceleration voltage was 80 kV. The aperture size of the condenser diaphragm was 200 µm. A lens diaphragm of 90 µm was used. TEM images were formed at different magnifications ranging from 15 to 200kX. **Figure** **1**A illustrates the revealed nanostructure of 0.25 wt%. MAM /PMMA blend. Thereafter, images such as Figure 1A are denoted by *u*(**x**), where *u* is the gray level intensity at every pixel **x**  = (*x*_1_,*x*_2_) .

In Figure 1A, nanostructures are contrasted by PTA with a strength that varies locally, i.e., some micellar objects contrast better with the background than some other nanostructures. Inhomogeneous and/or insufficient contrast ratio between phases is known to be a possible source of error in the automatic binarization (segmentation) of phases, e.g., Pinto et al.^[^ ^36^ ^]^ Of course, some would say that electronic micrographs typified by an inhomogeneous and/or insufficient contrast between objects (foreground) and the background may still be analyzed by manual methods^[^ ^37^, ^38^ ^]^ or hybrid methods combining automatic and manual image analysis procedures.^[^ ^36^ ^]^

#### 2.4.3 Cellular Structure

**Figure 2:**
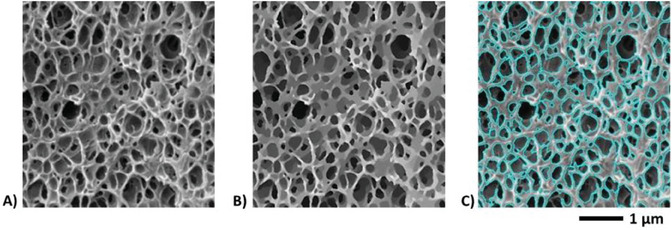
A) Local illustration of a foam produced from a PMMA/0.5 wt% MAM solid precursor saturated with scCO_2_ at 40 °C and 30 MPa and then foamed in one‐step batch foaming using an average PDR of 0.5 MPa s^−1^. B) Optimized image after applying to Figure 2A the nonlinear directional diffusive filter. The filtered image is a piecewise constant solution representing a simplified image at a lower resolution with sharper interfaces between voids and the solid lattice. C) In cyan, the segmented (labeled) cells superimposed upon the original image (Figure 2A).

The cellular structure of the foams was imaged at several magnifications (from 0.1 to 100 kX) with a scanning electron microscope (SEM) (a ThermoFisher Quanta 250 FEG SEM) at I2 m Laboratory (Bordeaux, France). For the observation, samples were fractured (perpendicularly to the direction of barrel injection) after cooling in liquid nitrogen. Samples were then coated with a gold layer of a few nanometers thick. SEM images were formed by collecting the secondary electron emission, e.g., **Figure** **2**A. In this instance, the contrast is said to be topographical and is made of three contributions: the slope contrast, the shading contrast, and the ridge contrast. The last two contributions promote foam cells that are darker than the polymeric background solid lattice. Yet, dependence of the secondary emission upon the slope contrast may work locally *the other way around*, depending on the local geometry of the sample. Thus, it is again recommended to further enhance contrast for the exact and automatic determination of the cell size distribution. To achieve that, the nonlinear directional diffusive process recalled above was used (Figure 2B). The subsequent automatic segmentation of foam cells standing out more clearly from a brighter solid lattice was achieved by simple thresholding of the gray level histogram (Figure 2C). Before thresholding, the sharpened images were if necessary corrected for shading. Cell sets segmented thereby (Figure 2C) were suited for the automatic determination of cell sizes after disconnecting overlapping pores.^[^ ^9^ ^]^

The cell number density was determined according to $N_{cell}={\left(\frac{m}{A}\right)}^{3/2}$, where *m* is the number of cells segmented in a (series of) image(s) and *A* is the area of the (series of) digital image(s) in cm^2^. The effective cell nucleation density *N*_0_ (effective nucleation points/cm^3^ or apparent number of bubbles nucleated per cm^3^ of the original unfoamed solid blend) was determined as:

(1) $$N_{0}=\frac{N_{cell}}{\frac{\rho_{f}}{\rho_{s}}}\;$$

The only assumption implicit in this widely used equation for *N*_0_ is that bubbles actually show an isotropic distribution. If the porosity ɛ is defined as $\varepsilon =N_{cell}\frac{\pi }{6}{\bar{D}}_{cell}^{3}$ with ${\bar{D}}_{cell}$ the average cell diameter (in cm) determined from 2D SEM micrographs, we have $N_{0}=\frac{N_{cell}}{\frac{\rho_{f}}{\rho_{s}}}\neq \frac{N_{cell}}{1-\varepsilon }$ for stereological reasons. The latter issue is certainly overlooked in the literature.

## 3 Results

### 3.1 Nanostructuration of the Acrylic Blends

**Figure 3:**
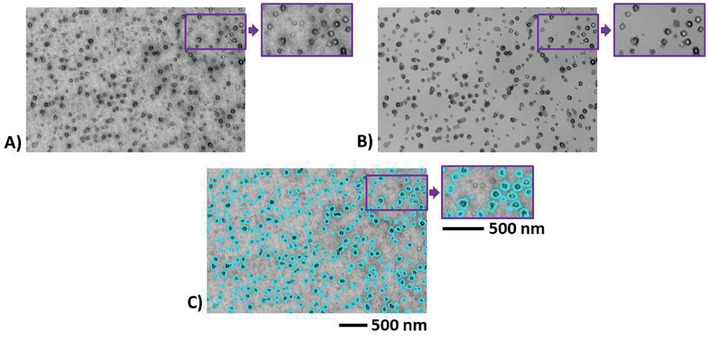
A) TEM micrograph (local area; magnification: 20 kX) showing the nanostructuration of a PMMA/MAM solid blend (10 wt% MAM). B) Filtered image at a coarser resolution representing a simplified image with discriminated sharper boundaries: the steeper edges of the surface nanostructures, preferentially colored by PTA, are selectively enhanced, while information relative to the thickness of the preparation is dissipated by blurring. C) Binarized counterpart of Figure 3B obtained by simple thresholding of the gray level histogram. The level set corresponding to the threshold value is superimposed in cyan upon the original image (Figure 3A).

As previously stated by Bernardo et al.^[^ ^12^ ^]^ from a very amount of MAM, TEM micrographs of the solid precursors treated by PTA revealed micellar‐like objects at the three amounts of MAM, e.g., Figure 1A at 0.25, 0.5 wt% MAM and **Figure** **3**A at 10 wt% MAM.

**Figure 4:**
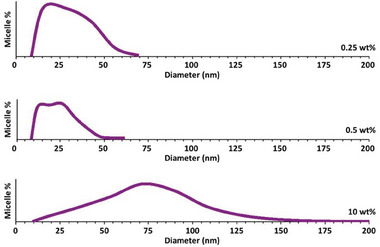
Size distributions of micellar objects automatically determined from binarized TEM micrographs, e.g., Figures 1 and 3. The finite size interval bins of the original discrete frequency histogram of the 2D nanostructure diameters are schematically represented as continuous distributions of the nanostructure percentile as a function of the nanostructure size. The vertical full scale is set to the highest micelle percentile of the original frequency histogram.

The micellar objects were automatically segmented after optimizing the TEM images according to the method described in Ref. [9] For robust statistics, several micrographs acquired at different magnifications were systematically binarized, enabling the calculation of the diameter of several thousand nanostructures for each sample. The size distributions thus obtained are given in **Figure** **4** for the three PMMA/MAM blends. The structural characteristics derived from these distributions, including the nano‐objects’ number density *N_object_*, the nano‐objects’ average diameter ${\bar{D}}_{object}$ and the aggregation number *N_aggregation_* (number of copolymer molecules per nanostructur*e*) are listed in **Table** **1**.

**Table 1:** Characteristics of PMMA/MAM solid precursors.

Solid PMMA/MAM blend ID	*N_objet_* : Nanostructure density [objects/cm^3^]	${\bar{D}}_{object}$: Nanostructure average diameter [nm]	ρ_*s*_: Density of the solid blends [g/cm^3^]	*N_aggregation_*: Aggregation number ^a)^
0.25 wt% MAM	6.3 10^13^ ± 0.4 10^13^	27 ± 1	1.19 ± 0.02	350
0.50 wt% MAM	1.0 10^14^ ± 0.1 10^14^	25 ± 2	1.18 ± 0.01	430
10.0 wt% MAM	2.4 10^14^ ± 0.2 10^14^	68 ± 8	1.19 ± 0.07	3700

^a)^ Assuming that all copolymers lie inside micellar objects, an upper bound of the aggregation number was estimated as $N_{aggregation}=\frac{w\;N_{a}\;\rho_{s}}{M_{n}\;N_{object}}$ where *w* is the amount of copolymer (wt%) and *N_a_* the Avogadro's number.

As exemplified in Figure 3A, staining by liquid diffusion produces mostly a surface selective coloration whereby the nanostructures intercepted by the thin slice surface (from which PTA rises) are preferentially contrasted. Nanostructures deeper‐lying in the preparation thickness would be hardly colored since nanostructures essentially do not percolate through the thickness of the slice. The image optimization method described above was accordingly advantageously used to selectively enhance, and segment, the darker surface nanostructures (Figure 3C). The nanostructures’ volumetric number density *N_object_* (number of nanosized domains/cm^3^) was consistently calculated using the stereological framework given in Equation (2).

(2) $$N_{object}={\left(\frac{n}{A}\right)}^{3/2}$$

Where *n* is the number of nanostructures segmented in an image and *A* is the area of the digital image in cm^2^.

The micellar object density *N_object_* decreases moderately as MAM content is reduced by almost two orders of magnitude (Table 1). As MAM content is reduced, nanostructures are also smaller (Table 1 and Figure 4). Following the reasoning of Bernardo et al.,^[^ ^12^ ^]^ the observed consistency between TEM observations (*N_object_* and ${\bar{D}}_{object}$) and the theoretical estimation of the aggregation number (based on the hypothesis that all the copolymers form micelles) supports that MAM lies essentially in nanostructures and does not remain solubilized in the PMMA matrix.

At 10 wt% MAM, the nanostructures’ size distribution spreads loosely over a decade of sizes (Figure 4). This contrasts with the narrower size distributions at lower MAM contents (Figure 4). Both the larger mean size and the larger dispersion of sizes contribute to the observed relatively low value of the object density *N_object_* at 10 wt% MAM (Table 1). Nevertheless, this low value is consistent with values reported by others, e.g., Pinto et al.^[^ ^33^ ^]^

**Figure 5:**
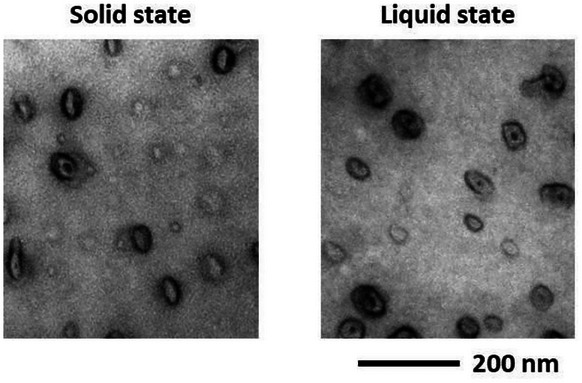
TEM micrographs showing (after staining) nanostructures in a PMMA/10 wt% MAM blend. Left: in the solid precursor (at 20 °C) after compounding in classical extrusion conditions/water cooling. Right: in the ≪ liquid ≫ state, i.e., after quenching the extrusion liquid rod in liquid nitrogen at the outlet of the extrusion die (180 °C).

These results confirm that the nanostructures, in a non‐equilibrium state, show a micellization. Yet TEM imagery does not provide here a clear‐cut answer as to the true inner structure of the micellar objects, i.e., I‐shaped versus U‐shaped BCP molecules. Even so, both I‐shaped and U‐shaped molecular conformations are expected to be efficient for localizing the CO_2_ toward providing a controlled number and a controlled spatial and instantaneous distribution of effective foaming nucleants. Last, **Figure** **5** suggests that MAM micellar nano‐objects are present even in the liquid state (“the melt”) within PMMA, i.e., after quenching the extrusion liquid rod in liquid nitrogen at the end of the extrusion die (180 °C) used for compounding the precursor blend. It supports the ability of MAM to generate similar nanosized micellar objects, whatever the conditions (this ability was also checked by comparing a single to a double extrusion blending; the same structures and the same foams were obtained.^[^ ^39^ ^]^ Such an illustration of structuration in both solid and ≪ liquid ≫ states is, to the best of our knowledge, shown for the first time.

### 3.2 Cellular Structures

The role of the scCO_2_ saturation temperature *T_sat_* upon the speed at which the foam vitrifies – thereby stopping cell growth and coalescence for achieving reduced cell dimensions – remains a complex issue, which is certainly overlooked in the literature. Indeed, as foaming is initiated by an essentially adiabatic pressure quench, the effective sample temperature *T_ef_* (*t*) is lower than *T_sat_* and generally unknown. Therefore, the vitrification speed – the key to achieve a nanosized porosity – depends both upon the time evolution of *T_ef_* (*t*) and the rise of the effective glass transition temperature of the plasticized system *T*_*g*, *ef*_(*t*). Recall that the system vitrifies as soon as *T*_*g*, *ef*_(*t*) >  *T_ef_* (*t*). The increase with time *t* of the effective glass transition temperature *T*_*g*, *ef*_(*t*) is linked to CO_2_ departure in response to cell nucleation and growth (the former is always disadvantaged by the latter if the two phenomena overlap over time). The cell nucleation rate per se (and the subsequent and/or synchronous cell growth and coalescence) depends also upon the BCP content and size, to which the dependence upon the PDR shall be added, e.g., Haurat et al.^[^ ^22^ ^]^

**Figure 6:**
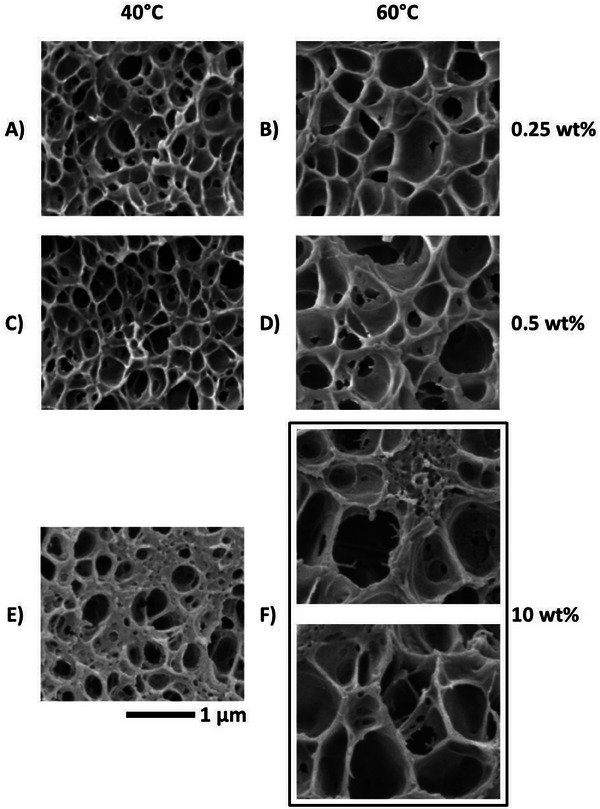
SEM images (local areas) showing the cellular structuration of porous materials produced from PMMA/MAM blends at a scCO_2_ saturation pressure of 30 MPa and at 40 °C (A, C, and E) or 60 °C (B, D, and F) with a subsequent average PDR of 0.5 MPa s^−1^ for foaming initiation. A and B: 0.25 wt% MAM; C and D: 0.5 wt% MAM; E and F: 10 wt% MAM. All images are at the same scale.

**Figure** **6** illustrates the cellular structure of the foams issuing from 0.25% to 10 wt% MAM blends. Figure 6 suggests an immediate first comment. At low MAM contents, the walls and struts of the solid skeleton are rather thin and the porosity is mostly monomodal. At higher MAM content (10 wt%), the struts and the walls are locally thick and clearly nanoporous. The porosity is bimodal with larger inter‐wall cells bounded by a porous solid lattice, which includes tiny intra‐wall nanosized closed cells (Figure 6E,F). More precisely, Figure 6E,F show well expanded local domains, i.e., local clusters of microsized cells (inter‐wall or inter‐skeleton pores) encapsulated by a thick solid skeleton including nanosized cells (inter‐wall or intra‐skeleton pores).

**Figure 7:**
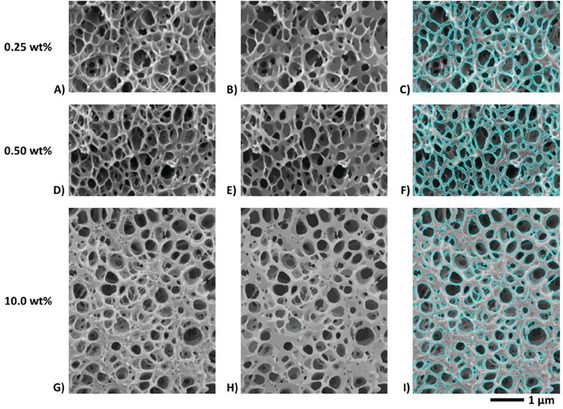
**First row**: Binarization of a SEM micrograph of a foam produced from a 99.75/0.25 PMMA/MAM blend at a scCO_2_ pressure of 30 MPa and at 40 °C for the saturation stage and using an average PDR of 0.5 MPa s^−1^ to trigger foaming. Left view: cellular structure (local zone). Middle view: filtered micrograph at a lower resolution showing enhanced contrast after running over the left view the method of image optimization recalled in Section 2.4. Right view: binary counterpart of the optimized image (middle view) after thresholding of the gray level histogram. The level set corresponding to the threshold value is superimposed in cyan upon the original image (left view). **Second row**: Idem for a foam from a 99.5/0.5 PMMA/MAM blend (*P_sat_*  =  30 MPa, *T_sat_*  =  40 °C and $\frac{\Delta P}{\Delta t}$  =  0.5 MPa s^−1^). **Third row**: Idem for a foam from a 90/10 PMMA/MAM blend (*P_sat_*  =  30 MPa, *T_sat_*  =  40 °C and $\frac{\Delta P}{\Delta t}$  =  0.5 MPa s^−1^). All images are at the same scale.

**Figure 8:**
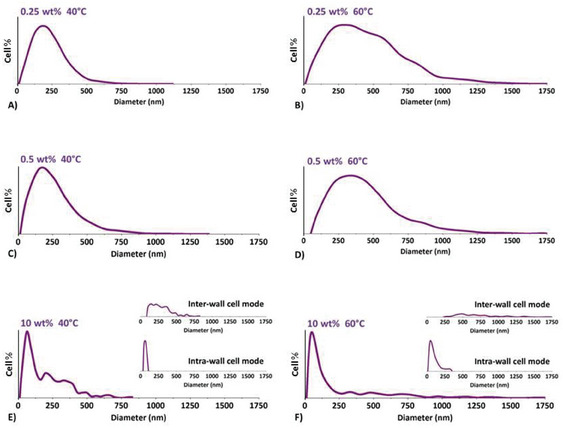
Cell size distributions of the foam samples are illustrated in Figure 6. The vertical full scale is set to the highest cell % of the original discrete frequency histogram. Foams produced from the 90/10 PMMA/MAM blends show two scales of porosity, with tiny intra‐wall cells of much smaller size than that of their inter‐wall counterparts; see Figure 6E,F for local illustrations. The derivation of the intra‐ and inter‐wall cell size modes in Figure 8E,F is addressed further below.

Bubbles were segmented as recalled in Section 2.4 and illustrated in **Figure** **7**. For robust statistics, several micrographs acquired at different magnifications were binarized for each sample. The calculated size distributions are given in **Figure** **8**. Characteristics derived from these size distributions such as the average cell size ${\bar{D}}_{cell}$, the cell number density *N_Cell_* (number of cells/cm^3^) and the apparent (or effective) nuclei density *N*_0_ are provided in **Table** **2**.

**Table 2:** Characteristics of PMMA/MAM foams.

Foam ID	Foam density ρ_*f*_ [g cm^−3^]	Average cell size ^a)^ ${\bar{D}}_{cell}$ [nm]	Cell density *N_Cell_* [cells cm^−3^]	Effective nuclei density ^b^ ^)^ *N*_0_ = *N_cell_* × ρ_*s*_/ρ_*f*_ [nuclei cm^−3^]
10% MAM, *T_sat_* = 40 °C	0.66 ± 0.01	193 ± 4	3.6 × 10^13^ ± 0.2 × 10^13^	6.5 × 10^13^ ± 0.8 × 10^13^
10% MAM, *T_sat_* = 60 °C	0.53 ± 0.01	239 ± 57	1.5 × 10^13^ ± 0.5 × 10^13^	3.4 × 10^13^ ± 1.4 × 10^13^
0.5% MAM, *T_sat_* = 40 °C	0.59 ± 0.02	264 ± 5	2.6 × 10^13^ ± 0.6 × 10^13^	5.2 × 10^13^ ± 1.4 × 10^13^
0.5% MAM, *T_sat_* = 60 °C	0.59 ± 0.02	445 ± 5	3.1 × 10^12^ ± 0.2 × 10^12^	6.2 × 10^12^ ± 0.3 × 10^12^
0.25% MAM, *T_sat_* = 40 °C	0.62 ± 0.01	234 ± 6	2.6 × 10^13^ ± 0.4 × 10^13^	5.0 × 10^13^ ± 0.9 × 10^13^
0.25% MAM, *T_sat_* = 60 °C	0.58 ± 0.01	461 ± 54	5.6 × 10^12^ ± 3.0 × 10^12^	1.1 × 10^13^ ± 0.6 × 10^13^

^a)^ For each sample, the average cell size ${\bar{D}}_{cell}$ and cell density *N_Cell_* were derived from a concatenated object set comprising thousands and even tens of thousands of cells. This large object set was built by merging *n* elementary object sets. Each elementary object set consisted of the set of cells segmented on a single SEM micrograph. Both for ${\bar{D}}_{cell}$ and *N_Cell_*, the uncertainty was assessed as an uncertainty on the mean, e.g., $\Delta \;{\bar{D}}_{cell}=\frac{\sigma }{\sqrt{n}}$ (where σ is the standard deviation calculated on the basis of *n* local mean values evaluated on a collection of *n* micrographs);

^b^ ^)^ The uncertainty in the effective nuclei density *N*_0_ was calculated by propagating errors in *N_cell_* and in the relative density $\frac{\rho_{f}}{\rho_{s}}$.

**Figure 9:**
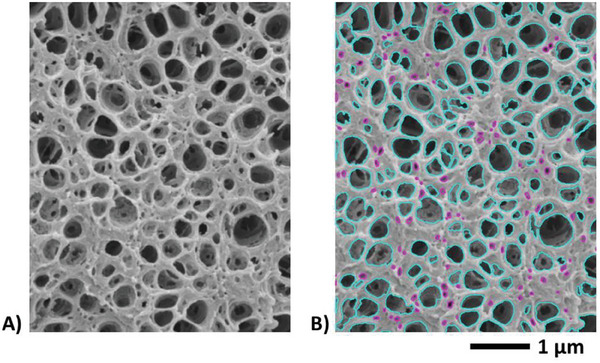
A) Cellular structuration of a foam produced at 40 °C from the 90/10 PMMA/MAM blend (scCO_2_ pressure: 30 MPa; average PDR: 0.5 MPa s^−1^). B) The segmented object set (cell set) (Figure 7I) was filtered by choosing the threshold value of an object size attribute (object diameter or area) that partitioned unequivocally the cell set in two classes of porosity: small‐sized intra‐wall cells (in magenta) versus much larger inter‐wall cells (in cyan). The threshold value was chosen from the object set overlaid on the image by selecting some of the largest cells within the walls and keeping as threshold value the largest size attribute value. See Demewoz and Yeh^[^ ^40^ ^]^ for a comparable filtering approach.

The intra‐ and inter‐wall cell size modes of the two bimodal size distributions (Figure 8E,F) were specified by filtering the segmented global object sets (bubble sets). Filtering consisted in removing those objects from an object set whose filter size attribute (object diameter or area) fell outside a specified range. See **Figure** **9** for a local illustration of this filter. The filtered cell sets determined over several micrographs were next pooled and diameter frequencies were converted to proportions, leading to the intra‐wall‐cell and inter‐wall‐cell size distributions given in Figure 8E,F. Average intra‐wall and inter‐wall cell sizes along with inter‐wall cell fraction (by number) derived from the two modes are listed in **Table** **3**.

**Table 3:** Two scales of porosity in foams from 90/10 PMMA/MAM: tiny‐sized intra‐wall cells versus larger inter‐wall cells.

Foam ID	Average intra‐wall cell size ${\bar{D}}_{intra-wall\;cell}$ [nm]	Average inter‐wall cell size ${\bar{D}}_{inter-wall\;cell}$ [nm]	Inter‐wall cell fraction [%] ^a)^
10% MAM, *T_sat_* = 40 °C	65 ± 5	277 ± 3	60 ± 1
10% MAM, *T_sat_* = 60 °C	95 ± 4	671 ± 18	25 ± 9

^a)^ The inter‐wall cell fraction was calculated as the inter‐cell number to total cell number ratio as illustrated in Figure 9.

## 4 Discussion

The development of nanosized polymeric foams by gas dissolution foaming requires (1) a high nuclei density and (2) mastering cell growth in such a way that coalescence is minimized^[^ ^15^, ^40^, ^41^, ^42^, ^43^, ^44^, ^45^ ^]^ According to Costeux,^[^ ^5^ ^]^ nanocellular CO_2_‐blown foams necessitate nuclei densities (*N*_0_) in the order of 10^15^–10^16^ cells cm^−3^ versus 10^9^–10^12^  cm^−3^ for microcellular foams. The values of *N*_0_ reached in this study are in the order of 1 × 10^13^ to 5 × 10^13^  cm^−3^ (Table 2). Therefore, our foam samples may be regarded as *intermediary* between microsized and nanosized porous materials.

### 4.1 Influence of the Saturation Temperature

The role of the saturation temperature *T_sat_* in acrylic polymers solid‐state (nonflowing) one‐step batch foaming is well known^[^ ^46^, ^47^, ^48^ ^]^ A lower saturation temperature, i.e., an increased CO_2_‐solubility^[^ ^49^ ^]^ optimizes the saturation step and shall promote nucleation. In this study, the effect of *T_sat_* follows this expected trend: a lowered *T_sat_* leads to an increased effective nuclei density *N*_0_ (Table 2).

**Figure 10:**
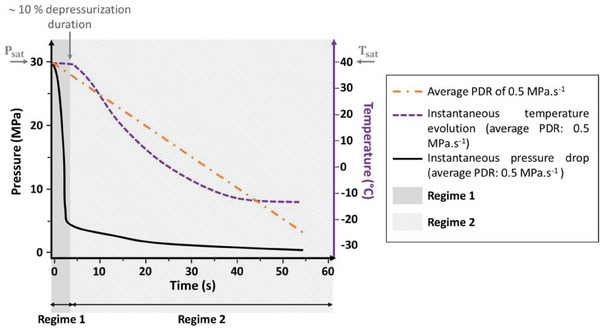
Hypothesis for a schematic representation of the instantaneous *true* pressure and temperature evolutions during a depressurization process of average PDR equal to 0.5 MPa s^−1^. Adapted from Haurat et al.^[^ ^22^ ^]^ and modified from original experimental work by Pinto et al.^[^ ^34^ ^]^ who used the same high‐pressure vessel (at LCPO Laboratory, Bordeaux, France) and the same operating conditions as in this study. The question risen by this scheme is which events occur or overlap in regime_1 and regime_2 (i.e N, G, vitrification, gas escape).

As *T_sat_* is lowered, all else being equal, the average cell size ${\bar{D}}_{cell}$ decreases (Table 2). Explaining this latter trend is a more complex issue in one‐step batch foaming than in two‐step batch foaming. In two‐step batch foaming, the foaming temperature is known and entirely independent of *T_sat_*. This is not the case in one‐step batch foaming where the material effective temperature during foaming is unknown and is related in an indefinite manner to *T_sat_* (see below). Part of the explanation of the decrease of ${\bar{D}}_{cell}$ as *T_sat_* is reduced is contained in the inverse correlation between *T_sat_* and effective nuclei density *N*_0_ recalled above. Yet the full explanation necessitates to establish the link between a lowered *T_sat_* and reduced cell growth and coalescence. Or put differently, the full explanation requires to establish the link between a lower *T_sat_* and a faster speed of vitrification. The speed at which the plasticized system vitrifies is determined by the interplay and competition between the kinetics of two variables: on the one hand, the effective glass transition temperature *T*_*g*, *ef*_(*t*) of the PMMA‐CO_2_ system and on the other hand, the sample effective temperature *T*_*ef*_(*t*). More precisely, the time after which *T*_*g*, *ef*_(*t*) >  *T*_*ef*_(*t*) (vitrification) depends upon the increasing evolution of *T*_*g*, *ef*_(*t*) and the decreasing evolution of *T*_*ef*_(*t*) during CO_2_ diffusion and foaming. Let us recall that pressure release, in one‐step batch foaming, induces a strong temperature drop in the foaming vessel (*T*_*ef*_(*t*) is lower than *T_sat_*) (**Figure** **10**). Note also that due to the plasticization effect of CO_2_, the effective glass transition temperature *T*_*g*, *ef*_ reaches an unknown value at the end of the saturation stage. This unknown value is most likely close to room temperature (vs≈115 °C before saturation)^[^ ^49^, ^50^, ^51^, ^52^, ^53^ ^]^ During pressure release and foaming, *T*_*g*, *ef*_(*t*) rises with time *t* due to the ongoing desorption/departure of CO_2_ in response cell nucleation and growth.

The decrease rate of *T*_*ef*_(*t*) is influenced by the initial value *T*_*ef*_ (0) = *T_sat_*  and by the instantaneous PDR, i.e., the decrease of the instantaneous pressure *P*(*t*) (Figure 10). The higher is the imposed average PDR, the lesser is the dependence of *T*_*ef*_(*t*) upon its initial value *T_sat_*.^[^ ^22^ ^]^ In this study, a moderate average PDR is used (0.5 MPa s^−1^). Consequently, the initial value of *T*_*ef*_(*t*), *T_sat_*, still impacts the kinetics of the decreasing *T*_*ef*_(*t*) and thereby constrains the speed at which *T*_*g*, *ef*_(*t*) is overcome (*T*_*g*, *ef*_(*t*) >  *T*_*ef*_(*t*)). This explains the observed (positive) correlation between *T_sat_* and the average cell size (the smaller the former is, the smaller is the latter; Tables 2 and 3).

At much higher average PDR, the situation would be different^[^ ^22^ ^]^ Indeed, the amplitude of the temperature drop that accompanies pressure release shall be far more important and faster. Thus *T_ef_* (*t*) shall reach values far below room temperature in the early stages of pressure release (*T_ef_* (*t*) is lower than *T*_*g*, *ef*_(*t*) at the early stages of pressure release). In this situation, one and/or the other of two hypotheses, yet unknown, may be advanced:

1. Only nucleation occurs during the early stages of pressure release, but foaming is prevented (the system is in the glassy state: *T_ef_* (*t*) <  *T*_*g*, *ef*_(*t*)). Then foaming initiates in the vessel only once *T_ef_* (*t*) crosses up *T*_*g*, *ef*_(*t*) around room temperature. Thus, we may consider that the foaming process, previously referred to as one‐step gas dissolution foaming, has more in common with a two‐step gas dissolution foaming process. Because *T*_*g*, *ef*_(*t*) rises quickly with cell growth, the system vitrifies again very quickly, quenching cell growth and coalescence much faster than in this study. In such a situation, a small spread of *T_sat_*, i.e., 20 °C (40 °C vs 60 °C), shall have no impact upon how fast cell growth and coalescence are blocked. This is to say *T_sat_* has no impact, only the kinetics of the rising *T*_*g*, *ef*_(*t*) plays the key role.
2. Both nucleation and limited cell growth have time to occur during the early stages of pressure release. However, the decrease of *T_ef_* (*t*) is so steep that a *T_sat_* spread of 20 °C (40 °C vs 60 °C) at the onset of pressure release has no impact upon the vanishing time lapse at which the system vitrifies. According to this second scenario, we are still in a one‐step gas dissolution foaming process, in contrast to scenario 1 above which is very much like to a two‐step gas dissolution foaming process.

As a first summary, a lower saturation temperature promotes nucleation and contributes to a reduced cell size^[^ ^5^, ^48^, ^54^, ^55^ ^]^ when a moderate average PDR is used. But previous studies presented as batch one‐step gas foaming and using high pressure drop rates with similar materials were most likely incurring in a two‐step approach.

### 4.2 Influence of MAM Content

Adding nanostructures to a homopolymer matrix aims at controlling the bubble number density and the uniformity of the bubble sizes. In other words, the expected result is to provide a controlled number and a controlled spatial and instantaneous distribution of effective nucleants promoting heterogeneous nucleation (over homogeneous nucleation). In this situation, porous cells can grow simultaneously toward a uniform size.

**Figure 11:**
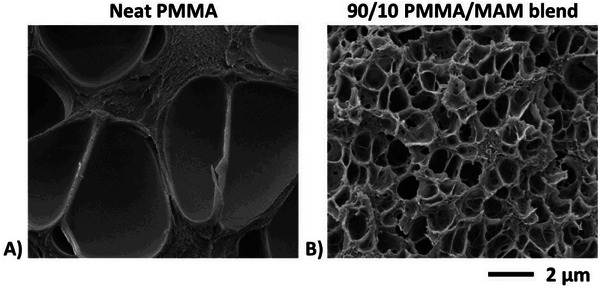
SEM images (same scale) showing the cellular structure of foams produced from neat PMMA (A) and from 90/10 PMMA/MAM blends (B) at a scCO_2_ pressure of 30 MPa and at 60 °C with an average PDR of 0.5 MPa s^−1^. The cell size distribution of the foam from neat PMMA (A) is given in Figure 12 and that of the foam from 90/10 PMMA/MAM blend (B) is provided in Figure 8F.

The strategy of BCP is already well proved to be efficient in two‐step batch and extrusion foaming processes. Prior to quantify the benefit brought by MAM addition in one‐step batch foaming, the structuration of a foam produced from neat PMMA is first qualitatively compared with that of a foam from PMMA/MAM blend (**Figure** **11**). The foam produced without block copolymers (Figure 11A) shows much larger cells than with BCP (Figure 11B). In the same line of two‐step gas dissolution batch foaming, addition of block copolymers in a well‐mastered (and simpler) one‐step batch foaming process is obviously a step in the targeted direction, i.e., decreasing porosity dimensions.

**Figure 12:**
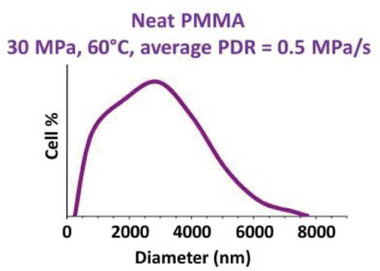
Cell size distribution of a foam produced from neat PMMA (Figure 11A). Refer to Table 4 for the main structural characteristics derived from this size distribution.

Our systems (one‐step batch foams from 40 or 60 °C) should be compared to previous similar studies, in order to drive new conclusions regarding foam generation. The large mean cell size (2892 nm) and low effective nuclei density (3.1 × 10^10^ nuclei cm^−3^) of the foam from neat PMMA (**Figure** **12** and **Table** **4**) may be viewed at variance with values reported by Pinto^[^ ^33^ ^]^ (at similar saturation pressure and average PDR), namely 90 nm and 2.9 × 10^15^ nuclei cm^−3^. The contradiction becomes only apparent when considering the low saturation temperature used by these authors, i.e., room temperature versus 60 °C in Figure 11A. The low temperature used by Pinto et al. places their work under either of the two scenarios provided above at the end of Section 4.1, whereby samples vitrify over a vanishingly small time lapse. In other words, use of a low saturation temperature has comparable effects to use of a very high average PDR. That is to say, for kinetic reasons, Pinto et al. using a lower saturation temperature with similar PMMA materials and procedures were most probably experiencing a two‐step procedure. Along that line, Pinto et al.^[^ ^33^ ^]^ stress that nanocellular structures can only be obtained from neat PMMA by combining high pressure, low temperature, and high PDR. Yet, for the kinetic reasons stated above, this method is very much like to be a two‐step gas dissolution foaming process. Still in this direction, microsized cellular structures from neat PMMA similar to that in Figure 11.A were observed by Reglero Ruiz et al.^[^ ^56^ ^]^ using operational conditions similar to ours, i.e., using an actual one‐step approach.

**Table 4:** Main characteristics of a foam from neat PMMA.

PMMA foam (Figure 11A)	Foam density ρ_*f*_ [g cm^−3^]	Average cell size ${\bar{D}}_{cell}$ [nm]	Cell density *N_Cell_* [cells cm^−3^]	Effective nuclei density *N*_0_ = *N_cell_* × ρ_*s*_/ρ_*f*_ [nuclei cm^−3^]
*P_sat_* = 30 MPa, *T_sat_* = 60 °C	0.6 ± 0.02	2892 ± 60	1.6 × 10^10^ ± 0.2 × 10^10^	3.1 × 10^10^ ± 0.5 × 10^10^

When using neat PMMA as solid precursor, scCO_2_ is uniformly sorbed in the plasticized homopolymer during saturation, which then hosts homogeneous nucleation upon pressure release. This invites nuclei and cell coalescence if the effective sample temperature *T_ef_* (*t*) remains long enough above the effective glass transition temperature *T*_*g*, *ef*_(*t*) during gas desorption and foaming. Such is the case in Figure 11A due to use of a moderate average PDR and a rather high saturation temperature (i.e., 60 °C to comply with the supercritical state). Cell growth and coalescence are reflected by the observed very large cell (Figure 11A and Table 4).

As stated by Reglero et al.^[^ ^57^ ^]^ in the tri‐block copolymer approach (Figure 11B), scCO_2_ is, there too, evenly sorbed in the PMMA matrix. Yet, it is also advantageously localized and concentrated in dispersed BCP nanostructures spaced apart from each other. In this situation, nucleation shall be primarily controlled by MAM nanostructuration. Several arguments can be raised in this direction:

1. At 0.25 wt% MAM and 40 °C, the effective nuclei density *N*_0_ (Table 2) and the nanostructure density *N_object_* (Table 1) are almost equal. In other words, one nanostructure leads to one bubble.
2. It can be argued that interfaces act as energetically favorable loci encouraging heterogeneous nucleation over homogenous nucleation^[^ ^58^, ^59^, ^60^, ^61^ ^]^ Even if interfaces in nanostructures formed by organic ABA copolymers such as MAM may not represent energetically favorable loci,^[^ ^62^ ^]^ it may still be argued that the neat PBA phase in nanostructures (whatever the exact and actually unknown structure of MAM nano‐objects is) shows a homogeneous Gibbs free energy barrier lower than that of neat PMMA.^[^ ^33^ ^]^

Consider now the influence of MAM content (and nanostructure sizes) upon the final cellular structure at the most favorable saturation temperature, i.e., *T_sat_* =  40°*C*:

The value of the effective nucleation density remains almost the same, ≈5 × 10^13^ nuclei cm^−3^, irrespective of MAM increment (Table 2). Use of 40 °C (lowest possible temperature to comply with the supercritical state) appears relatively high compared to other authors (*T_sat_* = 20 °C or even lower). This certainly delays vitrification of the surrounding PMMA matrix. As a consequence of the choice of 40 °C, the effective nuclei density in our work is tangibly lower than the value of 4.0 × 10^14^ reported by Pinto et al.^[^ ^33^ ^]^ at room temperature (also irrespective of MAM content). As recalled above, these authors using a lower *T_sat_* than in this study were most likely experiencing a two‐step procedure, while this study is using an actual one‐step approach.

Assume that in the bimodal sample (Figure 6E), the inter‐wall cells are the counterparts of the cells in monomodal samples (Figure 6A,C).^[^ ^64^ ^]^ Under this hypothesis, the average cell size produced from the three blends is nearly constant (Tables 2 and 3).

At *T_sat_* =  40°*C*, both effective nuclei density *N*_0_ and mean cell size are therefore nearly independent of MAM content, while the nanostructure density *N_object_* of the solid precursors increases with MAM increment (Tables 1, 2, 3). In other words, the ratio $\frac{N_{0}}{N_{object}}$ decreases with MAM increment. When homogenous nucleation is minimized, as is generally the case in the BCP approach,^[^ ^57^, ^58^, ^59^, ^62^, ^63^, ^64^ ^]^ this ratio can be viewed as the rate of effective nuclei. Its decrease with MAM increment can be explained as follows:

The use of a moderate average PDR results in a moderate nucleation rate. Therefore, nucleation spreads over time and overlaps with cell growth over a finite period of time. During the overlap time interval, nucleation (which is very sensitive to the blowing agent concentration) is disadvantaged by bubble growth which locally depresses the blowing agent. This behavior is all the more pronounced as MAM content is higher. Nanostructures are indeed grouped more closely (Figure 3). The first activated nucleants deplete CO_2_ near immediately adjacent nanostructures, which lowers the $\frac{N_{0}}{N_{objets}}$ ratio.^[^ ^63^ ^]^ This behavior is certainly exacerbated by the loose size distribution of the nanostructures in the solid precursor at 10 wt% (Figure 4). The early activated largest nucleants deplete locally CO_2_ around smaller potential nucleants. In addition, aggregated nanostructures at high MAM content are less efficient as they promote locally nuclei coalescence in the early stages of bubble growth. As nucleation spreads further over time due to the less favorable distribution of sizes at 10 wt%, nascent small bubbles are more often surrounded by larger bubbles which nucleated earlier in the initial stages of pressure release. According to Fick's first law, what happens to a nascent small cell depends on mass exchange of CO_2_ from/into the polymer into/from the cell^[^ ^5^, ^64^ ^]^ Zhu et al.^[^ ^64^ ^]^ showed numerically that the plasticized polymer gas concentration difference between the interface of small cells and that of larger cells causes a systematic ripening of the cell population by gas diffusion. Cell ripening designates « *the growth of larger cells from those of smaller sizes* ».^[^ ^64^ ^]^ Ripening further lowers the $\frac{N_{0}}{N_{objets}}$ ratio.

Therefore, the intensity of coalescence and/or ripening can actually be qualified through the value of the ratio $\frac{N_{0}}{N_{objets}}$.

At *T_sat_* =  60°*C*, the impact of MAM content upon the cellular structure reported above at *T_sat_* =  40°*C* is exacerbated by a larger cell size for reasons stated in Section 4.1. This larger cell size is consistent with trends reported elsewhere. For example, Pinto et al.^[^ ^34^ ^]^ observed a noticeable increase in mean cell size above a threshold saturation temperature of 50 °C.

### 4.3 On The Issue of Bimodal Cellular Structures

The bimodal porosity observed at 10 wt% of MAM (Figure 6E,F), is here explained by one of candidate mechanisms:

1. Nanostructures at 10 wt% MAM show a far larger distribution of sizes compared to the nanostructure populations at lower MAM contents (Figure 4). In the former case, nanostructures activate over a wider time interval. The largest nanostructures of the population have a lower energy of activation and are easily activated nucleants, which may contribute to the bimodal cell size distribution observed in Figure 6E,F ^[^ ^62^, ^63^ ^]^
2. Another explanation is connected to the kinetics of the depressurization stage (Figure 10). On several occasions, the pressure drop rate ($\frac{\Delta P}{\Delta t}=\frac{P_{sat}-P_{ambient}}{\Delta t}$, where Δ*t* is the time to return to *P_ambient_* after operating the on/off discharge valve) has been qualified as average PDR to stress that it is actually not controlled. Using an average PDR of 0.5 MPa s^−1^ in one‐step gas dissolution batch foaming of 90/10 PMMA/MAM with the same high‐pressure vessel as used in this study, Pinto et al.^[^ ^33^ ^]^ measured the instantaneous pressure *P*(*t*) (and temperature *T_ef_* (*t*)) during depressurization. During the early stages (during the first seconds) of depressurization, pressure decreases steeply at an instantaneous rate far higher (by almost one order of magnitude) than the average PDR (Figure 10). After this early stage, depressurization continues with a slow pressure decrease toward ambient pressure at a much slower instantaneous rate, say one order of magnitude lower than the average PDR (Figure 10). Arora et al.^[^ ^47^ ^]^ produced bimodal foams from a polystyrene precursor by reducing the pressure in two stages. The marked slope change in the instantaneous pressure decrease that typifies foaming experiments in this study (Figure 10) may be paralleled to a pressure reduction in two stages, leading to two successive and independent nucleation‐growth (N‐G) processes. As addressed in Haurat et al.^[^ ^22^ ^]^ the first N‐G process would be the usual one, responsible for the inter‐wall micro‐cell population (regime 1 in Figure 10). The second N‐G process, typified by less favorable instantaneous PDR, *T_ef_* and *T*_*g*,*ef*_ (regime 2 in Figure 10) would lead to a minimized cell growth in local areas where residual CO_2_ is still available in the PBA shell of micellar objects. This second N‐G process would be responsible for the intra‐wall nanosized bubble population. It shall be stressed that during the second regime (second N‐G process), the instantaneous PDR $\frac{dP(t)}{dt}$ is very slow, i.e., < 0.05 MPa s^−1^ and nearly asymptotic. At such a low PDR, a one‐step batch process would not induce foaming with neat PMMA after saturation at 40 or 60 °C.^[^ ^11^ ^]^ Therefore, it is quite unlikely that the nanosized intra‐wall cells be produced by homogenous nucleation in the homopolymer matrix.

Both the loose size distribution of the nanostructures at 10 wt% MAM (Figure 4) and the two‐fold dynamics of the instantaneous pressure and temperature during depressurization most likely jointly contribute to the bimodal porosity in foams from 90/10 PMMA/MAM. Conversely, the narrow size distributions of nanostructures at 0.25 and 0.5 wt% MAM contributes to the uniformity (mono‐modality) of foams produced from low MAM content precursors. So does the lower nanostructure number density *N_object_* at low MAM content (Table 1). Indeed, at low MAM content, the first N‐G process activates all available MAM nanostructures so that there is no MAM nucleants left for a second independent N‐G process. If one accepts this, the absence of two cell populations at low MAM content supports that homogeneous nucleation does not occur in series (regime 2; Figure 10) after heterogeneous nucleation (regime 1; Figure 10), whatever the MAM content is (10 wt% MAM included).

In brief, the low to moderate average PDRs ($\frac{\Delta P}{\Delta t})$ and the relatively high saturation temperatures (*T_sat_*), which typify actual batch one‐step gas foaming play a key role in the production of bimodal foams. In such operating conditions, nucleation spreads over time (nucleation and cell growth overlap); more precisely over the two different regimes of the decreasing instantaneous PDR ($\frac{dP(t)}{dt}$), responsible for two successive nucleation‐growth processes if the BCP content is high enough (typically 10 wt%). This is no longer true if the average PDR is significantly increased and/or *T_sat_* is too low.

In the latter cases, gas dissolution foaming is indeed most likely incurring in a two‐step‐like approach, more favorable to the production of « all nanosized » foams.

Bimodal foams, i.e., micro + nano foams, that can be accessed through an actual one‐step process, may be viewed as a smart alternative to « all nanosized » foams in the context of thermal insulation. Indeed, they include well expanded local clusters of micron‐sized cells; these well expanded local domains are encapsulated by a thick porous skeleton (walls and struts) containing closed nano‐sized cells. Thus, the conductive part of the effective thermal conductivity foams can benefit from the Knudsen effect within well expanded local domains showing micro‐sized cells. The Knudsen effect shall remain meaningful thanks to the poorly expanded nano porous thick solid skeleton encapsulating these local domains, and preventing thereby radiative thermal conduction from overriding the conductive component of the effective conductivity.^[^ ^22^ ^]^

For minimizing the conductive component by decreasing cell size and increasing the expansion ratio, a high number density of cells is required, leading to very thin struts and cell walls. Unfortunately, this ultimately increases the radiative transmittivity. Such increase of the radiative component often *erases* the performance gain (reduction of the conductive component) permitted by the Knudsen effect and a high gas fraction. This suggests that micro‐nano bimodal foams may represent better and easier candidates for improved thermal insulation than « all nanosized » foams. Though, this effect can be here minimized due to the low fraction of nano cells in the walls.

## 5 Conclusion

This work addresses a long‐overlooked facet of the gas dissolution approach to produce nanosized polymeric foams., i.e., the use of an *actual* one‐step procedure. It is first demonstrated that previous attempts using high PDR and/or low saturation temperatures, and presented as batch one‐step gas foaming, were most likely experiencing a two‐step procedure for kinetic reasons.

Then, the production of nanosized polymeric foams by gas dissolution actual/true one‐step batch foaming requires (**1**) a high effective nuclei density and (**2**) mastering cell growth so that coalescence is minimized. This work is essentially focused on the impact of the saturation temperature *T_sat_* and block copolymer content upon the above twofold requirement. This involves taking advantage of disposal of representative statistical size distributions for both the BCP nanostructures in the unfoamed precursor materials and the bubbles in the final foams. The calculation of these size distributions, i.e., the quantified input and output of the foaming process, is here largely facilitated by an upstream strategy of contrast enhancement (directional adaptive diffusive filtering) of object sets sampled in grey level digital images. Although the background of our approach is not new (widely used in the field of image restoration and denoising), application to polymer science is new. This approach is used as a method for *virtually reconstructing* interfaces and interphases, thereby providing *new image reconstructions*.

Conclusions dealing with organic scCO_2_‐induced polymer foams are the following: Using a moderate average PDR (0.5 MPa s^−1^) to trigger foaming, the decreasing kinetics of the system effective temperature *T*_*ef*_(*t*) (due to the adiabatic pressure quench) remains influenced by its initial value *T_sat_* =  *T*_*ef*_(*t*  =  0) . Thus, the lower is *T_sat_*, the shorter is the time lapse *t* at which the rising effective glass transition temperature *T*_*g*, *ef*_(*t*) of the plasticized system (due to ongoing gas desorption) crosses up *T*_*ef*_(*t*), i.e., *T*_*g*, *ef*_(*t*) >  *T*_*ef*_(*t*) and the system enters the glassy state. This explains the (positive) correlation between *T_sat_* and the average bubble size (the smaller cells, the lower saturation temperature).

Both effective nuclei density *N*_0_ and mean cell size are shown to be nearly independent of MAM content over the scrutinized range (0.25% to 10 wt% MAM), while the nanostructure density *N_object_* of the solid precursor increases with MAM increment. Therefore, the ratio $\frac{N_{0}}{N_{object}}$ (ratio of effective nucleants) decreases with MAM increment. This behavior is explained by the moderate nucleation rate which follows from the use of a moderate average PDR. Consequently, nucleation and cell growth overlap and the former is penalized by the latter. This behavior is intensified by the loose size distribution of nanostructures, which typifies the solid precursor blend at 10 wt% MAM.

Quite interestingly, foams produced from 90/10 PMMA/MAM blends (high MAM content) show a bimodal porosity with microsized inter‐wall cells bounded by a porous solid lattice which includes intra‐wall closed nanosized cells. This distinctive feature is shown to be the consequence of (1) a larger distribution of nanostructure sizes at high MAM content and (2) the two‐fold kinetics of the system instantaneous temperature *T*_*ef*_(*t*) and pressure *P*(*t*) decrease during pressure release. The latter, which can be paralleled to a pressure reduction in two stages, gives rise to two consecutive independent nucleation‐growth (N‐G) processes responsible for the two bubble populations. The monomodal cell sizes observed at lower MAM contents (0.25 and 0.5 wt% MAM) support that the two bubble populations observed at 10 wt% MAM are initiated by heterogeneous nucleation without input from homogeneous nucleation in the homopolymer PMMA matrix.

Finally, it is argued that bimodal foams, i.e., micro/nanostructured foams, which can be accessed through an actual one‐step process, may represent a smart alternative to ≪ all nanosized ≫ foams in the context of thermal super insulation.

## Conflict of Interest

The authors declare no conflict of interest.

## Data Availability

The data that support the findings of this study are available from the corresponding author upon reasonable request.
